# Forskolin Inhibits Lipopolysaccharide-Induced Modulation of MCP-1 and GPR120 in 3T3-L1 Adipocytes through an Inhibition of NFκB

**DOI:** 10.1155/2016/1431789

**Published:** 2016-11-02

**Authors:** Jeanne Durendale Chiadak, Tatjana Arsenijevic, Kevin Verstrepen, Françoise Gregoire, Nargis Bolaky, Valérie Delforge, Véronique Flamand, Jason Perret, Christine Delporte

**Affiliations:** ^1^Laboratory of Pathophysiological and Nutritional Biochemistry, Université Libre de Bruxelles, Brussels, Belgium; ^2^Department of Biochemistry, Faculty of Sciences, University of Dschang, Dschang, Cameroon; ^3^Institute for Medical Immunology, Faculty of Medicine, Université Libre de Bruxelles, Gosselies, Belgium

## Abstract

In an obese state, Toll-like receptor-4 (TLR-4) upregulates proinflammatory adipokines secretion including monocyte chemotactic protein-1 (MCP-1) in adipose tissue. In contrast, G-protein coupled receptor 120 (GPR120) mediates antiobesity effects. The aim of this study was to determine the signaling pathway by which Forskolin (FK), a cyclic adenosine monophosphate- (cAMP-) promoting agent causing positive changes in body composition in overweight and obese adult men, affects MCP-1 and GPR120 expression during an inflammatory response induced by lipopolysaccharide (LPS) in adipocytes, such as in an obese state. 3T3-L1 cells differentiated into adipocytes (DC) were stimulated with LPS in the absence or presence of FK and inhibitors of TLR-4 and inhibitor of kappa B (I*κ*B*α*). In DC, LPS increased MCP-1, TLR-4, and nuclear factor-*κ*B1 (NF*κ*B1) mRNA levels, whereas it decreased GPR120 mRNA levels. In DC, FK inhibited the LPS-induced increase in MCP-1, TLR-4, and NF*κ*B1 mRNA levels and the LPS-induced decrease in GPR120 mRNA. BAY11-7082 and CLI-095 abolished these LPS-induced effects. In conclusion, FK inhibits LPS-induced increase in MCP-1 mRNA levels and decrease in GPR120 mRNA levels in adipocytes and may be a potential treatment for inflammation in obesity. Furthermore, TLR-4-induced activation of NF*κ*B may be involved in the LPS-induced regulation of these genes.

## 1. Introduction

In addition to storing excess energy, adipose tissue is now widely recognized as an important endocrine organ. Indeed, it secretes numerous cytokines called “adipokines” which have various functions, including macrophage recruitment, regulation of feeding behavior, energy homeostasis, and insulin sensitivity [1]. Obesity is characterized by adipose tissue inflammation and macrophage infiltration [2–4]. Adipose tissue expression of monocyte chemotactic protein-1 (MCP-1) and circulating MCP-1 levels are increased upon obesity in rodent, suggesting that MCP-1-mediated macrophage infiltration of adipose tissue may contribute to the metabolic disequilibrium associated with obesity and insulin resistance [5, 6]. Evidence providing an inflammatory link between obesity and type 2 diabetes is accumulating. In numerous animal and clinical studies, obesity is associated with a state of low-grade, chronic inflammation in liver and adipose tissue, which includes activation of the innate immune system and the appearance of proinflammatory immune cells [7, 8]. Nutritional strategies designed to alleviate adipokines dysregulation include modulating dietary fatty acids, with the majority of evidence suggesting the anti-inflammatory effects of the marine derived long-chain n-3 polyunsaturated fatty acids (PUFA) eicosapentaenoic acid (20:5n-3, EPA) and docosahexaenoic acid (22:6n-3, DHA) which can blunt lipopolysaccharide- (LPS-) stimulated elevations in adipokines [9–13]. Furthermore, activation of G-protein coupled receptor 120 (GPR120) by DHA antagonizes the proinflammatory effects of tumor necrosis factors-alpha (TNF-*α*) and lipopolysaccharide in a macrophage cell line [14]. The inhibitory effects of DHA on LPS-Toll-like receptor-4- (TLR-4-) induced activation of nuclear factor-kappa B (NFkB) activity [15, 16] might be mediated by a GPR120 and *β*-Arrestin 2-dependent mechanism [14]. *β*-Arrestin proteins play an important role in regulating the responsiveness of G-protein coupled receptors (GPCRs) by contributing to mechanisms involved in both GPCR desensitization and resensitization [17].

Previous studies suggest that saturated fatty acids promote inflammation by activating TLR-4 on adipocytes and macrophages [18]. Indeed, mice lacking TLR-4 are protected against high-fat diet-induced obesity, inflammation, and insulin resistance because they are resistant to the suppression of insulin signaling during lipid infusion and exhibit reduced insulin-mediated changes in systemic glucose metabolism [19]. Forskolin (FK) is a labdane diterpene, isolated from the roots of the* Coleus forskohlii* plant, a perennial herb with fleshy fibrous roots belonging to the mint family of plants. In the early-to-mid-1980s, Forskolin was primarily used as an agent to help a number of cardiovascular disease conditions, mainly through a vasodilator effect [20]. This effect resulted from increased adenylate cyclase activity within the body. Forskolin causes positive changes in body composition in overweight and obese adult men [21]. One of the potential explanations for the decrease in fat mass and body fat percentage may be adenylate cyclase activation and, thus, cyclic adenosine monophosphate (cAMP) accumulation within adipose tissue, which stimulates free fatty acid release and lipolysis. Indeed, Forskolin has been widely used as a potent activator of adenylate cyclase in cellular preparations to study cAMP-dependent transduction pathways [22–25]. As circulating MCP-1 levels are increased in rodent obesity and the role of Forskolin in fat mass reduction is clearly established, the aim of this study was to determine on one hand the expression of MCP-1, TLR-4, GPR120, *β*-Arrestin 2, and NF*κ*B1 expression in DC and on the other hand the signaling pathway by which Forskolin affects MCP-1 and GPR120 expression in the LPS-induced inflammatory response in 3T3-L1 cells differentiated into adipocytes.

## 2. Materials and Methods

### 2.1. Reagents

Dulbecco's modified Eagle's medium (DMEM, 4.5 g/L glucose), streptomycin/penicillin, fetal bovine serum, horse serum, and calf serum were provided by Invitrogen (Carlsbad, CA, USA). Bovine insulin, 3-isobutyl-1-methylxanthine (IBMX), dexamethasone, lipopolysaccharides, and Forskolin were purchased from Sigma (St. Louis, MO, USA). TLR-4 signaling inhibitor (CLI-095) and kappa B (I*κ*B*α*) inhibitor (BAY11-7082) were purchased from InvivoGen (San Diego, CA, USA). Anti-*β*-actin and anti-I*κ*B*α* came from Millipore (Temecula, CA, USA), and anti-phospho-I*κ*B*α* (Ser32/36) (5A5) was purchased from Cell Signaling Technology, Inc. (Danvers, MA, USA).

### 2.2. Cell Culture and Treatments

3T3-L1 murine preadipocyte cells were grown in DMEM supplemented with 10% calf serum, 200 U/mL penicillin, and 200 U/mL streptomycin in 8% CO_2_ humidified atmosphere at 37°C until confluence. Two days after confluence, to induce adipocyte differentiation, cells were incubated for 60 h in DMEM supplemented with 10% fetal bovine serum and containing 500 *µ*M IBMX, 0.25 *µ*M dexamethasone, and 10 *µ*g/mL insulin. The cells were then maintained in the culture medium supplemented with insulin only and this media was changed every 2 days (day 5 and day 7) until complete differentiation (monitored by lipid droplet accumulation under the microscope and confirmed by Oil Red Coloration) had occurred (day 9). On day 9, the differentiated 3T3-L1 cells (DC) were treated for 4 h with water followed by 4 h with water and ethanol (CTL) or 4 h with water and 4 h with 10 *µ*M Forskolin (FK) or 4 h with 1 *µ*g/mL LPS followed by 4 h with LPS and ethanol (LPS) or 4 h with LPS followed by 4 h LPS and FK (LPS + FK). Both cells and culture media from cultured undifferentiated cells (UDC; at day 0) and from DC (at day 9 following treatment) were harvested. To determine how the expressions of the genes of interest are modulated by NF*κ*B activation, the DC were treated with I*κ*B*α* inhibitor BAY11-7082 at 10 *µ*M. The TLR-4 signaling inhibitor CLI-095 at 3 *µ*M was also used. For inhibitor studies, DC were pretreated with the inhibitor for 1 hour before exposure to LPS stimulation and then coincubated with LPS and the inhibitor for 4 h prior to RNA extraction and 24 h prior to protein extraction.

### 2.3. RNA Isolation

Isolation of RNA, as well as assessment of RNA concentration and purity, and RNA integrity were performed as previously described [26, 27].

### 2.4. RT-qPCR

Design of qPCR primers, cDNA synthesis, and qPCR reactions were performed as previously described [26]. The primer pairs which were used are shown in Table 1.

### 2.5. Analysis of Gene Expression Stability

Gene expression stability analysis and matching statistics were performed using Biogazelle qBASE Plus software [28]. Data were normalized using the references genes, tyrosine 3-monooxygenase/tryptophan 5-monooxygenase activation protein, zeta polypeptide (mmYwhaz), non-POU-domain containing octamer binding protein (mmNONO), and *β*-actin (mmACTB), which were previously validated for this cellular and experimental system [26].

### 2.6. MCP-1 ELISA Assay

MCP-1 protein levels of the culture media were determined by ELISA using a Duoset ELISA kit from R&D Diagnostics (Minneapolis, MN, USA).

### 2.7. Protein Extraction and Western Blot Analysis

DC were washed with calcium- and magnesium-free PBS and lysed in 1 mL of lysis buffer containing 50 mM Tris/HCl (pH 7.5), 150 mM NaCl, 0.5% Nonidet P40, 50 mM NaF, 1 mM sodium orthovanadate, dithiothreitol, and a cocktail of protease inhibitors (cOmplete EDTA-free, Roche). Whole cell lysates were prepared and submitted to SDS-polyacrylamide gel electrophoresis (SDS-PAGE) in the presence of 5%  *β*-mercaptoethanol using 12% polyacrylamide gels. Harvest of DC and preparation of whole cell lysates were performed as previously described [27]. Proteins were transferred to polyvinylidene difluoride membranes and immunolabeled using primary antibodies against I*κ*B*α*, phospho-I*κ*B*α*, and *β*-actin. The bound primary antibodies were detected using secondary anti-mouse or anti-rabbit antibodies (GE Healthcare, Little Chalfont, Buckinghamshire, UK) and ECL chemiluminescence detection kit (PerkinElmer, Waltham, MA, USA). The protein bands were scanned and digitized, and the density of each band was determined using the Quantity One software (Bio-Rad Laboratories, Hercules, CA, USA).

### 2.8. Statistical Analysis

Data are presented as mean ± SEM of 3 experiments. Group means were compared by paired *t*-test and *t*-test for unique sample. Differences were considered statistically significant at *p* < 0.05. All statistical analyses were performed using SPSS 22 (IBM Corp. version 22.0.0.0).

## 3. Results

### 3.1. Expression of MCP-1, TLR-4, GPR120, and *β*-Arrestin 2 mRNA Levels in UDC and DC

**Figure 1:**
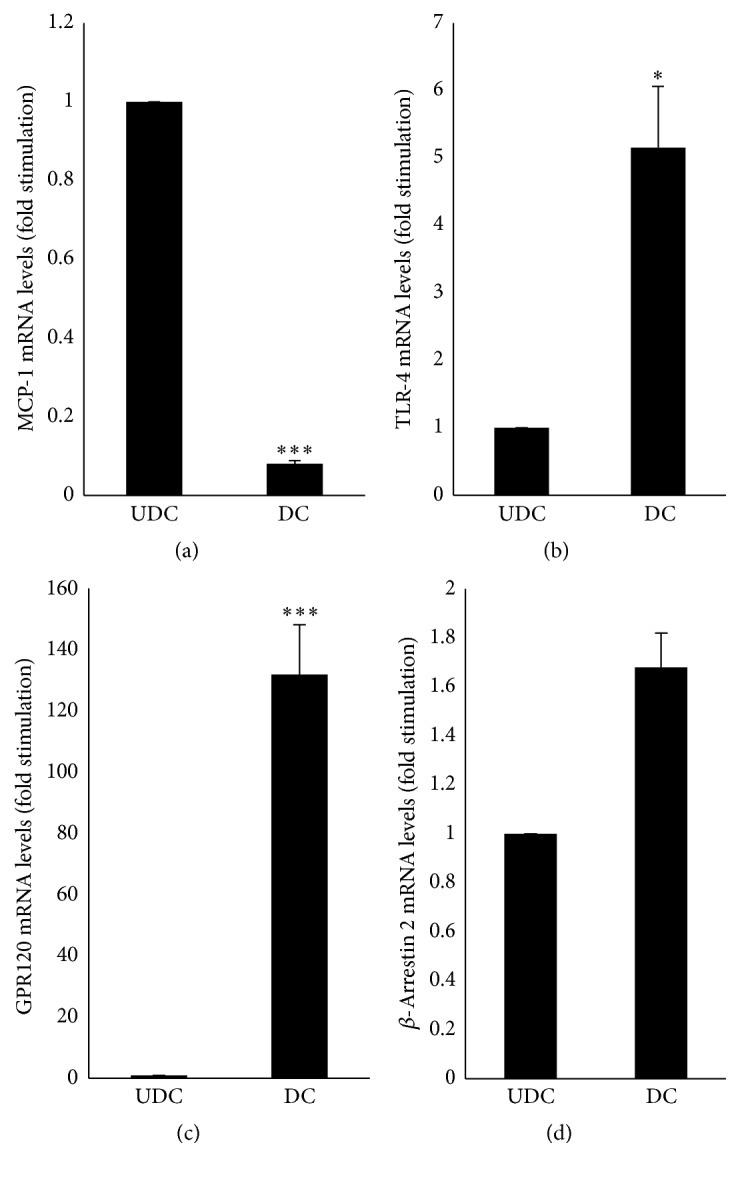
Expression of mRNA of MCP-1 (a), TLR-4 (b), GPR120 (c), and *β*-Arrestin 2 (d) in undifferentiated versus differentiated 3T3-L1 cells. UDC and DC were obtained as described under Materials and Methods. The results are expressed as relative mRNA levels (fold stimulation over UDC set to 1) and are the means ± SEM of 3 independent experiments. Data were analyzed by *t*-test for unique sample; ^*∗*^ *p* < 0.05 and ^*∗∗∗*^ *p* < 0.005 versus UDC.

MCP-1, TLR-4, GPR120, and *β*-Arrestin 2 mRNA levels were measured by RT-qPCR in UDC and DC. Upon adipocyte differentiation, TLR-4, GPR120, and *β*-Arrestin 2 mRNA levels were significantly upregulated 5-fold (*p* < 0.05), 130-fold (*p* < 0.005), and 1.6-fold (*p* < 0.05), respectively (Figure 1). In contrast, MCP-1 mRNA levels were significantly downregulated 0.08-fold (*p* < 0.005) upon adipocyte differentiation (Figure 1).

### 3.2. Effect of FK and LPS Modulation of MCP-1 mRNA and Protein Levels in DC

**Figure 2:**
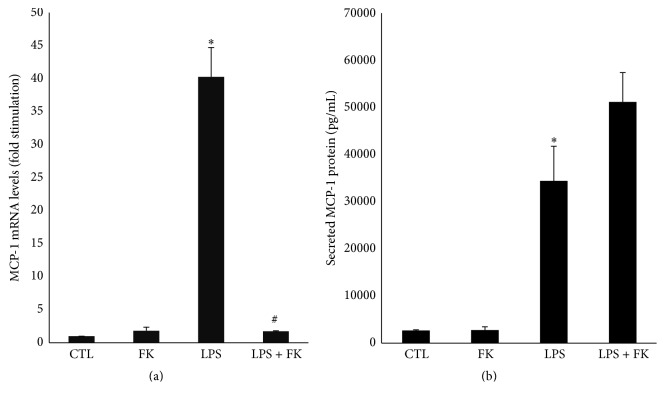
mRNA expression of MCP-1 (a) and MCP-1 protein secretion (b) in differentiated 3T3-L1 cells treated with LPS in the presence or absence of FK. Differentiated 3T3-L1 cells were treated as described in Materials and Methods under the following conditions: CTL, 10 *µ*M FK, 1 *µ*g/mL LPS, and LPS + FK. (a) The results are expressed as mRNA levels (fold stimulation over CTL set to 1) and (b) secreted MCP-1 protein levels are the means ± SEM of 3 independent experiments. Data were analyzed using *t*-test for unique sample and paired *t*-test; ^*∗*^ *p* < 0.05 versus CTL; ^#^ *p* < 0.05 versus LPS.

In DC, LPS significantly upregulated both mRNA (40-fold; Figure 2(a)) and protein (12.8-fold, Figure 2(b)) levels of MCP-1 (*p* < 0.05), as compared to CTL. In DC, FK did not significantly modify both MCP-1 mRNA and protein levels as compared to CTL (Figure 2). Upon treatment of DC with both LPS and FK, the MCP-1 mRNA level was significantly decreased by 95.7% as compared to LPS-treated DC (*p* < 0.05, Figure 2(a)). However, MCP-1 protein level was not significantly modified under LPS and FK treatment as compared to LPS treatment alone at the time points assessed (Figure 2(b)).

### 3.3. Effect of FK and LPS on TLR-4 mRNA Levels in DC

**Figure 3:**
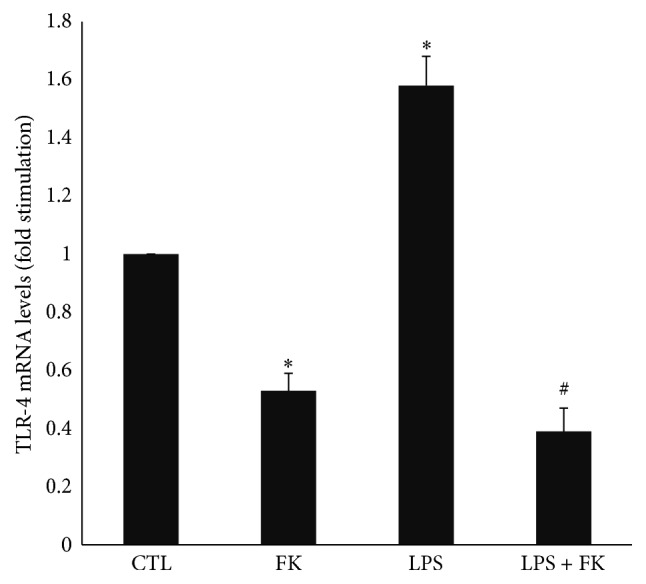
mRNA expression of TLR-4 in differentiated 3T3-L1 cells treated with LPS in the presence or absence of FK. Differentiated 3T3-L1 cells were treated as described in Materials and Methods under the following conditions: CTL, 10 *µ*M FK, 1 *µ*g/mL LPS, and LPS + FK. The results are expressed as mRNA levels (fold stimulation over CTL set to 1) and are the means ± SEM of 3 independent experiments. Data were analyzed using *t*-test for unique sample and paired *t*-test; ^*∗*^ *p* < 0.05 versus CTL; ^#^ *p* < 0.05 versus LPS.

LPS significantly increased TLR-4 mRNA level (1.6-fold) as compared to CTL (*p* < 0.05), while FK significantly decreased it (0.5-fold; *p* < 0.05) (Figure 3). TLR-4 mRNA level was significantly decreased by 73.5% in response to LPS and FK treatment as compared to LPS treatment (*p* < 0.05) (Figure 3).

### 3.4. Effect of FK and LPS on GPR120 mRNA Levels in DC

**Figure 4:**
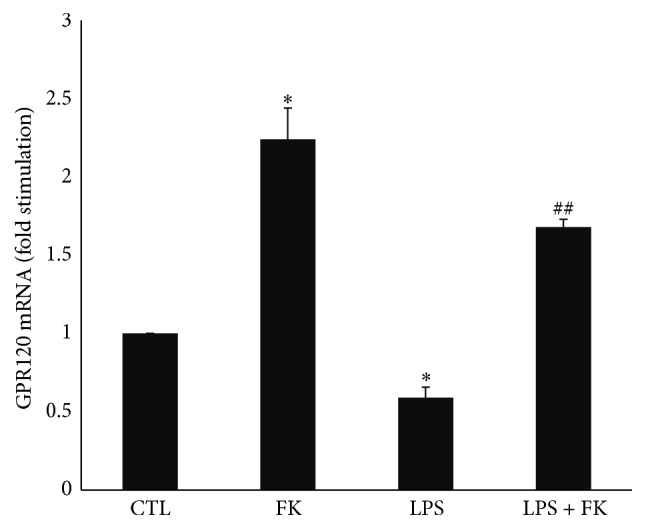
mRNA expression of GPR120 in differentiated 3T3-L1 cells treated with LPS in the presence or absence of FK. Differentiated 3T3-L1 cells were treated as described in Materials and Methods under the following conditions: CTL, 10 *µ*M FK, 1 *µ*g/mL LPS, and LPS + FK. The results are expressed as mRNA levels (fold stimulation over CTL set to 1) and are the means ± SEM of 3 independent experiments. Data were analyzed using *t*-test for unique sample and paired *t*-test; ^*∗*^ *p* < 0.05 versus CTL; ^##^ *p* < 0.01 versus LPS.

LPS significantly decreased GPR120 mRNA level (0.6-fold) as compared to CTL (*p* < 0.05), while FK significantly increased it (2.2-fold; *p* < 0.05) (Figure 4). LPS and FK significantly increased 2.8-fold the GPR120 mRNA level as compared to LPS treatment (*p* < 0.01) (Figure 4).

### 3.5. Effect of FK and LPS on *β*-Arrestin 2 mRNA in DC

**Figure 5:**
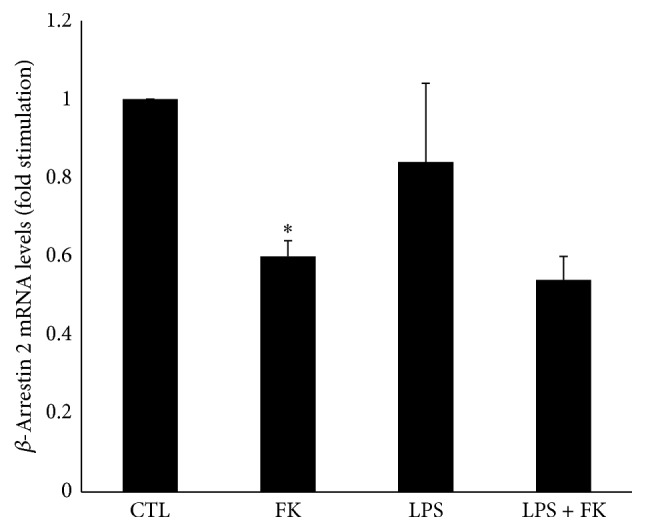
mRNA expression of *β*-Arrestin 2 in differentiated 3T3-L1 cells treated with LPS in the presence or absence of FK. Differentiated 3T3-L1 cells were treated as described in Materials and Methods under the following conditions: CTL, 10 *µ*M FK, 1 *µ*g/mL LPS, and LPS + FK. The results are expressed as mRNA levels (fold stimulation over CTL set to 1) and are the means ± SEM of 3 independent experiments. Data were analyzed using *t*-test for unique sample and paired *t*-test; ^*∗*^ *p* < 0.05 versus CTL.

LPS had no effect on *β*-Arrestin 2 mRNA level, whereas FK treatment significantly decreased it (0.6-fold; *p* < 0.05) (Figure 5). In addition, *β*-Arrestin 2 mRNA level was not significantly modified under LPS and FK treatment as compared to that of LPS treatment alone (Figure 5).

### 3.6. Effects of LPS and FK on NF*κ*B1 mRNA and I*κ*B*α* Protein Levels in DC

**Figure 6:**
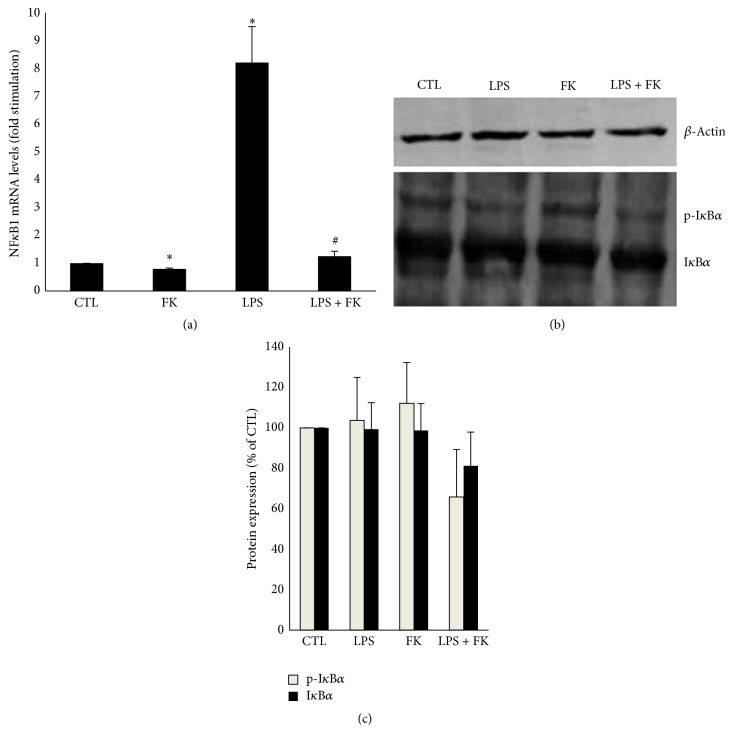
NF*κ*B1 mRNA and I*κ*B*α* protein levels in differentiated 3T3-L1 cells treated with LPS in the absence or presence of FK. Differentiated 3T3-L1 cells were treated as described in Materials and Methods under the following conditions: CTL, 10 *µ*M FK, 1 *µ*g/mL LPS, and LPS + FK. (a) The results are expressed as mRNA levels (fold stimulation over CTL set to 1). (b) Phospho-I*κ*B*α* and total I*κ*B*α* protein levels were determined in DC by Western blot analysis. (c) Semiquantitative determination of protein expression was performed as described under Materials and Methods. The results are expressed as protein expression (ratio of total I*κ*B*α* or P-I*κ*B*α* band density over *β*-actin band density) expressed as percent of the CTL value (CTL set to 100%) and are the mean ± SEM (*n* = 3) (in % of CTL). Data were analyzed using *t*-test for unique sample and paired *t*-test; ^*∗*^ *p* < 0.05 versus CTL; ^#^ *p* < 0.05 versus LPS.

LPS have been shown to stimulate secretion of inflammatory adipokines through activation of Toll-like receptor-4 (TLR-4) and the downstream transcription factor nuclear factor, NF*κ*B, in adipocytes [29–31]. In addition, a small increase in cytosolic concentrations of I*κ*B*α* negatively affects NF*κ*B nuclear translocation [32]. We therefore examined the effect of LPS and FK on NF*κ*B1 mRNA levels and I*κ*B*α* protein levels in DC and the result shows that the NF*κ*B1 mRNA level was significantly upregulated in the presence of LPS as compared to CTL (*p* < 0.05). FK significantly decreased by 87.5% the NF*κ*B1 mRNA level as compared to that of LPS-treated DC (*p* < 0.05) (Figure 6(a)). To determine if Forskolin affected I*κ*B*α* proteolysis and thereby NF*κ*B1 activation, total and phosphorylated I*κ*B*α* protein levels were analyzed by semiquantitative Western blotting (Figures 6(b) and 6(c)). In DC treated with and without LPS in the presence or absence of Forskolin, no significant differences were observed in terms of I*κ*B*α* (total and phosphorylated) protein level (Figures 6(b) and 6(c)).

### 3.7. Effects of a TLR-4 Inhibitor on MCP-1, GPR120, TLR-4, and NF*κ*B1 mRNA Levels upon LPS Treatment

**Figure 7:**
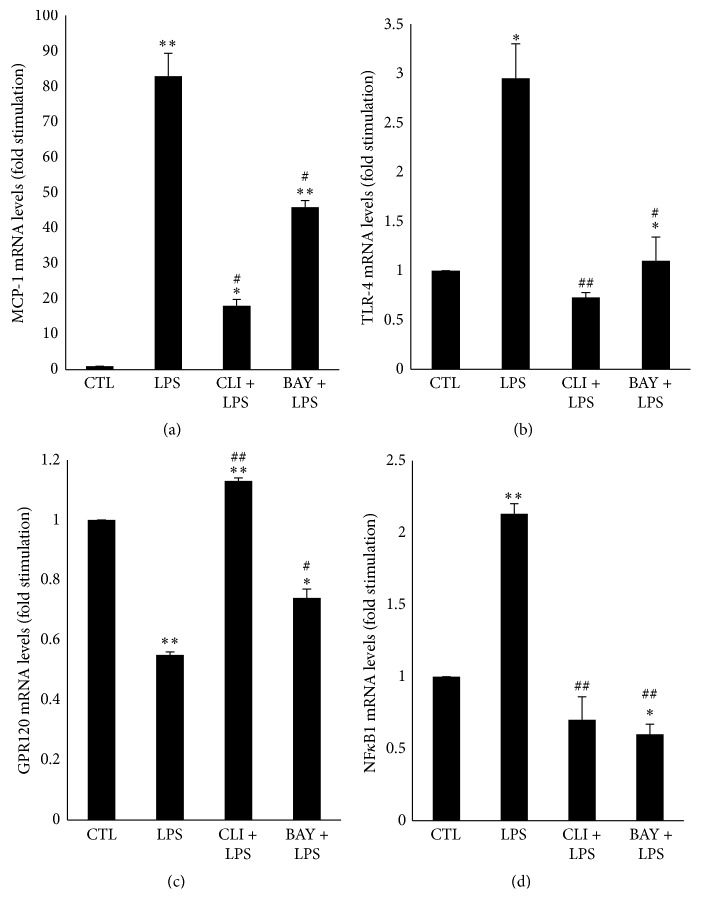
mRNA expression of MCP-1 (a), TLR-4 (b), GPR120 (c), and NF*κ*B1 (d) in differentiated 3T3-L1 cells treated with LPS in the presence or absence of inhibitors. Differentiated 3T3-L1 cells were treated as described in Materials and Methods under the following conditions: CTL, 1 *µ*g/mL LPS, 3 *µ*M CLI-095 + 1 *µ*g/mL LPS (CLI + LPS), and 10 *µ*M of BAY11-7082 + 1 *µ*g/mL LPS (BAY + LPS). The results are expressed as mRNA levels (fold stimulation over CTL set to 1) and are the means ± SEM of 3 independent experiments. Data were analyzed using *t*-test for unique sample and paired *t*-test; ^*∗*^ *p* < 0.05; ^*∗∗*^ *p* < 0.01 versus CTL; ^#^ *p* < 0.05; ^##^ *p* < 0.01 versus LPS.

To determine whether the regulation of MCP-1, GPR-120, and TLR-4 mRNA levels induced by LPS was mediated by TLR-4, a LPS receptor, DC were preincubated for 1 hour in the absence or presence of a TLR-4 inhibitor (3 *µ*M of CLI095) prior to a 4 h treatment with LPS (1 *µ*g/mL). The MCP-1 mRNA level was significantly increased by LPS (80-fold; *p* < 0.05) as compared to CTL, while CLI095 significantly decreased it by 80% (*p* < 0.05) (Figure 7). LPS decreased the GPR120 mRNA (0.6-fold; *p* < 0.05) as compared to CTL, while CLI095 abolished it (*p* < 0.05) (Figure 7). In addition, both TLR-4 and NF*κ*B1 mRNA levels were significantly upregulated upon LPS treatment (3-fold and 2-fold, resp.; *p* < 0.05) as compared to CTL, but they were abolished in the presence of CLI095 (Figure 7).

### 3.8. Effects of an I*κ*B*α* Inhibitor on MCP-1, GPR120, TLR-4, and NF*κ*B1 mRNA Levels upon LPS Treatment

To specifically assess the involvement of an NF*κ*B1-dependent pathway in the regulation of MCP-1 and GPR120 mRNA levels induced by LPS, DC were pretreated with an I*κ*B*α* inhibitor, BAY11-7082, prior to LPS treatment. The LPS-induced increase in MCP-1 mRNA level was significantly decreased by 54% by BAY11-7082 (*p* < 0.05) (Figure 7). The LPS-induced decrease in GPR120 mRNA level was abolished by BAY11-7082 (*p* < 0.05) (Figure 7). LPS-induced increase in both TLR-4 and NF*κ*B1 mRNA levels was abolished in the presence of BAY11-7082 (Figure 7).

## 4. Discussion

Obesity is strongly associated with increased risk of cardiovascular disorders. Recent studies have shown that increased levels of proinflammatory cytokines, including MCP-1, are involved in obesity and insulin resistance [4, 33] and that excess intake and endogenous release (lipolysis) of saturated fatty acids might enhance expression of TLR-4 target genes including MCP-1. Indeed, TLR-4-deficient knockout mice fed with a diet rich in saturated fatty acid had lower macrophage infiltration and MCP-1 expression in their visceral adipose tissue as compared to wild-type mice [34]. LPS have been shown to stimulate secretion of inflammatory adipokines through activation of Toll-like receptor-4 (TLR-4) and the downstream transcription factor nuclear factor, NF*κ*B, in adipocytes [29–31]. In addition, Forskolin caused positive changes in body composition in overweight and obese adult men [21]. In the present study, we analyzed for the first time the effect of Forskolin on LPS-induced modulation of MCP-1, TLR-4, GPR120, *β*-Arrestin 2, and NF*κ*B1 gene expression.

We confirmed that MCP-1 mRNA and proteins levels were increased following LPS treatment in 3T3-L1 cells differentiated into adipocytes and that this effect was mediated by TLR-4, as a TLR-4 inhibitor abolished the effect of LPS. Furthermore, we showed for the first time that Forskolin can inhibit the LPS-induced increase of MCP-1 mRNA level. Although MCP-1 protein levels were also increased in response to LPS, Forskolin did not significantly affect this LPS-induced increase in MCP-1 protein levels. Direct relationship between the mRNA and protein levels is not always observed, as the levels of both mRNA and proteins levels result from a ratio between synthesis and degradation. In addition, half-lives of proteins within cells vary widely from minutes to several days [35]. As both MCP-1 mRNA and secreted MCP-1 protein levels were not determined at similar times following cell treatment (4 hours for mRNA and 24 hours for proteins), distinct incubation times and the obvious distinct effects of FK on mRNA and protein half-lives (i.e., affecting synthesis and/or degradation) are likely to account for the observed data. Further work will include kinetic studies to determine the half-lives of mRNA and protein.

**Figure 8:**
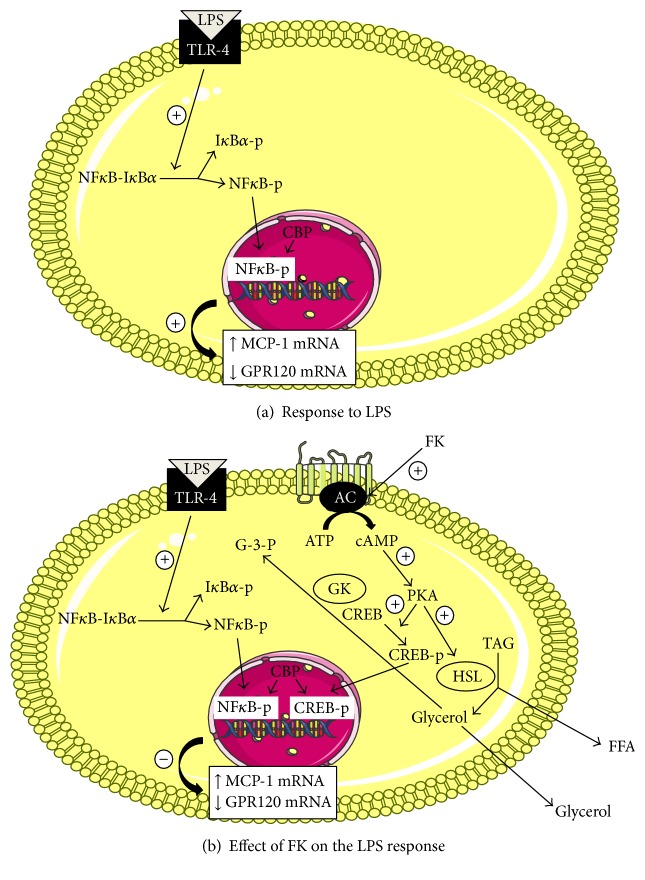
Data summary. (a) When adipocytes are stimulated with LPS, GPR120 mRNA levels are decreased, while MCP-1 mRNA and protein levels are upregulated via TLR-4 signaling pathway and NF*κ*B activation. (b) When adipocytes are treated with LPS in the presence of FK, the LPS-induced effects on both MCP-1 and GPR120 are inhibited via PKA and CREB activation necessitating CBP binding. AC: adenylyl cyclase; ATP: adenosine triphosphate; cAMP: cyclic adenosine monophosphate; CBP: CREB-binding protein; CREB: cAMP response element-binding protein; FFA: free fatty acids; FK: Forskolin; G-3-P: glycerol-3-phosphate; GK: glycerol kinase; GPR120: G-protein coupled receptor 120; H: catecholamines or hormones; HSL: hormone sensitive lipase; I*κ*B*α*: inhibitor of kappa B; LPS: lipopolysaccharide; MCP-1: monocyte chemotactic protein-1; NF*κ*B: nuclear factor-*κ*B; p: phosphorylated; PKA: protein kinase A; TAG: triacylglycerol; TLR-4: Toll-like receptor 4.

Our data suggest that Forskolin might therefore represent an interesting approach to decrease inflammation related to obesity in adipose tissue. The signaling pathway involved in the inhibition of LPS-induced MCP-1 mRNA levels by Forskolin was also investigated. Toll-like receptors, expressed on virtually all human cells and binding a wide spectrum of exogenous and endogenous ligands such as bacterial LPS, are involved in metabolic disorders [36, 37]. Our result confirmed that LPS alone increased TLR-4 mRNA levels in differentiated adipocytes and this increase was abolished by CLI095, a TLR-4 inhibitor. These data suggest a positive feedback loop between LPS and TLR-4. Furthermore, our data are in agreement with those showing that TLR-4 knockout mice are protected against high-fat diet-induced obesity, inflammation, and insulin resistance and exhibited reduced insulin-mediated changes in systemic glucose metabolism [19]. Our data also show for the first time that Forskolin can inhibit LPS-induced increase in TLR-4 mRNA levels. These data clearly suggest that Forskolin could play a protective role in the inflammatory environment of adipose tissue. In addition, our study originally reported that Forskolin increased GPR120 mRNA level both in the absence and in the presence of LPS, that is, a proinflammatory cue. These data are particularly interesting as, in a macrophage cell line, DHA (a GPR120 agonist) antagonizes the proinflammatory effects of TNF-*α* and LPS and requires the presence of GPR120 to counteract the proinflammatory actions of LPS on cytokines' gene expression and protein secretion [14]. In the presence of LPS, DHA significantly decreased MCP-1 mRNA level but increased both *β*-Arrestin 2 and GPR120 mRNA levels [38]. The inhibitory effects of DHA on LPS-(TLR-4)-induced activation of NFkB activity might be mediated by a GPR120-*β*-Arrestin 2-dependent mechanism [14]. We hypothesized that the effect of Forskolin on LPS-induced increase of MCP-1 mRNA level could involve a GPR120-*β*-Arrestin 2-dependent anti-inflammatory mechanism requiring NF*κ*B activation via I*κ*B*α* degradation upon phosphorylation. Thereby, we examined the effect of an I*κ*B*α* inhibitor, BAY11-7082, on MCP-1, GPR120, TLR-4, and NF*κ*B1 mRNA levels. We observed that BAY11-7082 abolished the effect of LPS on MCP-1, GPR120, TLR-4, and NF*κ*B1 mRNA levels; this confirmed the involvement of an NF*κ*B pathway in the regulation of expression of these genes. In addition, our results showed that Forskolin alone or in the presence of LPS inhibits NF*κ*B1 mRNA levels in DC. Furthermore, no change in both total and phosphorylated I*κ*B*α* protein levels could be detected by semiquantitative Western blot analysis upon adipocyte treatment with LPS and Forskolin alone or in combination. Recent reports have hypothesized that small increases in cytosolic concentrations of I*κ*B*α* negatively affect NF*κ*B nuclear translocation [32]. This hypothesis relies on the fact that only 10% of NF*κ*B is translocated to the nucleus at a steady state. Therefore, even a 2-fold increase in I*κ*B*α* protein concentration might be sufficient to retain the entire NF*κ*B activity in the cytoplasm [32]. Other studies have shown that Forskolin inhibits NF*κ*B-mediated transcription of several genes and that various doses of Forskolin did not significantly reduce the proteolytic degradation of I*κ*B*α* in HUVEC and THP-1 cells [39]. On the contrary, Forskolin increased the cytoplasmic levels of I*κ*B*α* in Jurkat T-cells, which selectively decreases the nuclear translocation of p65 [40], while another report [41] has shown that *β*-agonists exert their anti-inflammatory effects by increasing the cytoplasmic concentrations of I*κ*B*α* in monocytic cells. It should be noted that these authors did not investigate the production of I*κ*B*α* at the same time points and in the same cell types. The free I*κ*B*α* has a short half-life in vivo [42, 43], supporting the idea that dissociation from NF-kB leads to rapid degradation of I*κ*B*α*. In contrast to its remarkable stability when bound to NF*κ*B, free I*κ*B*α* is intrinsically very unstable, its half-life is <10 min, and it is rapidly degraded in a process that does not require phosphorylation or ubiquitination [44, 45]. This rapid degradation of I*κ*B*α* could explain why, in our study, no changes in I*κ*B*α* protein levels (total and phosphorylated) could be detected following 24 h incubation with Forskolin in the absence or presence of LPS. Even if the effects of Forskolin on the I*κ*B*α* protein level differ between studies [39–41], including ours, we showed that Forskolin exerts its anti-inflammatory effects by inhibiting the expression of NF*κ*B-dependent genes. In our study, we observed that LPS-induced increases in MCP-1, TLR-4, and NF*κ*B mRNA levels were downregulated by Forskolin. On the other hand, GPR120 mRNA levels were upregulated in the presence of Forskolin. GPR120 plays an anti-inflammatory role by activating anti-inflammatory pathways [14]. Our data clearly reveal an interaction between NF*κ*B and cAMP pathways in adipocytes as Forskolin exerts its anti-inflammatory role in adipocytes by inhibiting NF*κ*B-mediated gene transcription. Such inhibition could result from the binding competition between cAMP response element-binding protein (CREB) and NF*κ*B to the coactivator CREB-binding protein (CBP), a protein necessary for efficient gene transcription [46]. Indeed, upon activation of the protein kinase A (PKA) pathway, increased phosphorylated CREB-CBP complexes will form, inducing an inhibition of NF*κ*B (p65) [46]. PKA activation reduces the induction of a distinct set of genes in monocytes and endothelial cells by inhibiting NF*κ*B-mediated gene transcription [47]. Therefore, to account for the data observed in our study, we hypothesize that Forskolin exerts its anti-inflammatory effects on LPS-induced modulation of NF*κ*B target genes in adipocytes by increasing cAMP levels, PKA activity, CREB phosphorylation, and CREB interaction with CBP (Figure 8). Additional studies will be required to assess the involvement of PKA and CREB activation, as well as interaction between CBP, CREB, and NF*κ*B in such process.

In conclusion, pharmacological agents that elevate intracellular levels of cAMP, such as Forskolin, may be useful for the treatment of inflammation associated with obesity.

## Figures and Tables

**Table 1:** Real-time PCR primer sequences.

Gene	Forward primer (5′⇒3′)	Reverse primer (5′⇒3′)
mmNONO	TGCTCCTGTGCCACCTGGTACTC	CCGGAGCTGGACGGTTGAATGC
mmACTB	CCTGTGCTGCTCACCGAGGC	GACCCCGTCTCCGGAGTCCATC
mmYwhaz	AAAAACAGCTTTCGATGAAGCC	GCCGGTTAATTTTCCCCTCC
mmMCP-1	TTCACCAGCAAGATGATCCCA	TCCTTCTTGGGGTCAGCACA
mmTLR-4	AGGACTCTGATCATGGCACTG	GGAATGTCATCAGGGACTTTGC
mmGPR120	GGTGCCGGGACTGGTCATTGT	AGAGCGTGCGGAAGAGTCGGT
mm*β*-Arrestin 2	ATGGGAGAAAAACCCGGGAC	CACAGGGTCCACTTTGTCCA
mmNF*κ*B1	CTGCAGCTCTTACCCTGGAG	GTAATTGCGTGGCAGAGTGG
